# Development of a high-resolution detection module for the INSERT SPECT/MRI system

**DOI:** 10.1186/2197-7364-1-S1-A24

**Published:** 2014-07-29

**Authors:** Paolo Busca, Carlo Fiorini, Arslan D Butt, Michele Occhipinti, Riccardo Quaglia, Paolo Trigilio, Gabor Nemeth, Peter Major, Tamas Bukki, Kalman Nagy, Claudio Piemonte, Alessandro Ferri, Alberto Gola, Jan Rieger, Thoralf Niendorf

**Affiliations:** Politecnico di Milano, Dipartimento di Elettronica, Informazione e Bioingegneria, Via Golgi 40, 20133 Milano, Italy; Istituto Nazionale di Fisica Nucleare, Sezione di Milano, Via Celoria 16, 20133 Milano, Italy; Mediso Medical Imaging Systems, Alsotorokvesz 14, H-1022 Budapest, Hungary; Fondazione Bruno Kessler (FBK), Via Sommarive, 18, 38123 Trento, Italy; MRI.TOOLS GmbH, Robert-Roessle-Str. 10, 13125 Berlin, Germany; Berlin Ultrahigh Field Facility (B.U.F.F.), Max-Delbrueck-Center for Molecular Medicine, Berlin, Germany

A new multi-modality imaging tool is under development in the framework of the INSERT (Integrated SPECT/MRI for Enhanced Stratification in Radio-chemo Therapy) project, supported by the European Community. The final goal is to develop a custom SPECT apparatus that can be used as an insert for commercially available MRI systems. INSERT is expected to offer more effective and earlier diagnosis with potentially better outcome in survival for the treatment of brain tumors, primarily glioma. Two SPECT prototypes are being developed, one dedicated to preclinical imaging (7 and 9.4 T), the second one dedicated to clinical imaging (3 T).

The fundamental unit is a 5 cm x 5 cm gamma camera, based on the well-established Anger architecture with a continuous CsI:Tl scintillator readout by an array of silicon photomultipliers (SiPMs). The photodetector matrix will be composed by 12x12 SiPMs (FBK), each one with an active area of 4x4 mm^2^, for an overall field of view of 50.40x51.70 mm^2^, considering also insensitive areas between different detectors. In order to reduce complexity and costs the 144 channels are shortcut in group of 4 and readout by a custom-designed 36 channels ASIC. Each electronic channel features a fast current conveyor stage, followed by an RC filter with selectable peaking times and the electronics necessary to provide an appropriate output for the data acquisition system.

Preliminary Monte Carlo simulations suggest a spatial resolution between 0.8 and 1 mm and an energy resolution between 11% and 15% (140 keV), depending on the dark count rate of the SiPM technology (100-500 kHz/mm^2^). Experimental measurements are under development to confirm these results. For example, a single 4x4 SiPM (FBK, RGB-HD), coupled to a CsI:Tl scintillator, has been readout by a single channel version of the ASIC, providing an energy resolution close to 12% at 122 keV at room temperature.

